# Increased Concentrations of Glutamate and Glutamine in Normal-Appearing White Matter of Patients with Multiple Sclerosis and Normal MR Imaging Brain Scans

**DOI:** 10.1371/journal.pone.0061817

**Published:** 2013-04-17

**Authors:** Anders Tisell, Olof Dahlqvist Leinhard, Jan Bertus Marcel Warntjes, Anne Aalto, Örjan Smedby, Anne-Marie Landtblom, Peter Lundberg

**Affiliations:** 1 Center for Medical Image Science and Visualization (CMIV), Linköping University, Linköping, Sweden; 2 Radiation Physics, Department of Medical and Health Sciences (IMH), Linköping University, Department of Radiation Physics UHL, County Council of Östergötland, Linköping, Sweden; 3 Clinical Physiology, IMH, Linköping University, Linköping, Sweden; 4 Radiology, IMH, Linköping University, Department of Radiology, Västervik Hospital, Linköping, Sweden; 5 Radiology, IMH, Linköping University, Department of Radiology UHL, County Council of Östergötland, Linköping, Sweden; 6 Neurology, Department of Clinical and Experimental Medicine (IKE), Division of Neuroscience, Linköping University, Neurology Clinic LiM, County Council of Östergötland, Linköping, Sweden; St. Jude Children's Research Hospital, United States of America

## Abstract

In Multiple Sclerosis (MS) the relationship between disease process in normal-appearing white matter (NAWM) and the development of white matter lesions is not well understood. In this study we used single voxel proton ‘Quantitative Magnetic Resonance Spectroscopy’ (qMRS) to characterize the NAWM and thalamus both in atypical ‘Clinically Definite MS’ (CDMS) patients, MRI_neg_ (N = 15) with very few lesions (two or fewer lesions), and in typical CDMS patients, MRI_pos_ (N = 20) with lesions, in comparison with healthy control subjects (N = 20). In addition, the metabolite concentrations were also correlated with extent of brain atrophy measured using Brain Parenchymal Fraction (BPF) and severity of the disease measured using ‘Multiple Sclerosis Severity Score’ (MSSS). Elevated concentrations of glutamate and glutamine (Glx) were observed in both MS groups (MRI_neg_ 8.12 mM, *p*<0.001 and MRI_pos_ 7.96 mM *p*<0.001) compared to controls, 6.76 mM. Linear regressions of Glx and total creatine (tCr) with MSSS were 0.16±0.06 mM/MSSS (*p = *0.02) for Glx and 0.06±0.03 mM/MSSS (*p = *0.04) for tCr, respectively. Moreover, linear regressions of tCr and *myo*-Inositol (*m*Ins) with BPF were −6.22±1.63 mM/BPF (*p*<0.001) for tCr and −7.71±2.43 mM/BPF (p = 0.003) for *m*Ins. Furthermore, the MRI_pos_ patients had lower N-acetylaspartate and N-acetylaspartate-glutamate (tNA) and elevated *m*Ins concentrations in NAWM compared to both controls (tNA: *p = *0.04 *m*Ins *p*<0.001) and MRI_neg_ (tNA: *p = *0.03 , *m*Ins: *p = *0.002). The results suggest that Glx may be an important marker for pathology in non-lesional white matter in MS. Moreover, Glx is related to the severity of MS independent of number of lesions in the patient. In contrast, increased glial density indicated by increased *m*Ins and decreased neuronal density indicated by the decreased tNA, were only observed in NAWM of typical CDMS patients with white matter lesions.

## Introduction

Multiple sclerosis (MS) is often described as a multi-focal inflammatory and demyelinating disease of the central nervous system (CNS) [1], and it is characterized by lesions in the white matter where demyelination, axonal transection, astrocytic proliferation and oligondendrocytic loss have been found [2]. However, MS is a condition that has several subtypes, differentiated both regarding clinical development [1] and pathology [3]. Moreover, pathological changes besides white matter lesions have been found in gray matter in both cortical [4] as well as in deep gray structures [5], and in normal-appearing white matter (NAWM) [6].

White matter lesions are often clearly visible on conventional MRI images, and active inflammation can be visualized using contrast-enhanced MRI. In contrast, cortical lesions and pathologies in NAWM are not visible using conventional MRI; thus the radiological diagnosis of MS is based mainly on characterizing white matter lesions only [7], [8], [9]. Clearly, the formation of a lesion implies a disease process, but the number and extent of white matter lesions have poor correlation both with long-term clinical outcome and the continuous process of neurodegeneration in MS [1], [10]. In contrast, brain atrophy has been shown to correlate closely with disease progression [11], [12]. Nevertheless, there are still many issues on the cause of variations and patterns of both clinical symptoms and pathology in MS that remain to be solved, with the aims of determining both prognosis and personalized, effective, and individualized treatment.

Proton magnetic resonance spectroscopy (^1^H-MRS) which is tool for investigating MS *in vivo* has been used for investigating NAWM in MS patients. The results have been inconclusive in regard to some details [13]. One explanation could be that total creatine is often used as an ‘internal reference’, in spite of its observed variability in MS [13], [14] and also with age [15]. This clearly points to the importance of using quantitative magnetic resonance spectroscopy (qMRS). QMRS measurements of NAWM in ‘clinically definite MS’ (CDMS) patients have shown increased glutamate concentration (Glu) [16], increased total creatine concentration (tCr) [13], [14], increased *myo*-Inositol concentration (*m*Ins) [14], and decreased total N-acetylaspartate concentration (tNA) [13]. Due to the low spatial resolution of MRS (ca. 1 cc) it is difficult to investigate cortical gray matter using MRS. However, it is possible to use MRS in deep gray matter structures, and increased *m*Ins and decreased tNA have previously been found in the thalamus [17].

In a recent report we implemented a method for qMRS based on internal water scaling and calibration with ‘quantitative magnetic resonance imaging’ (qMRI) [18]. The qMRI volume can also be used for calculating brain parenchymal fraction (BPF) which can be used for assessment of brain atrophy [19].

In a minority of MS patients, no lesions at all are detected using conventional MRI, which makes these patients an interesting model for investigating MS pathology. These patients include a group for whom the clinical investigation must be extraordinarily thorough before it leads to the diagnosis of atypical MS. Our research has paid particular interest to this group of patients, for example in a study by Gustafsson *et al.* [20] where patients with CDMS and no radiologically visible lesions were observed, the patients showed no decreases in tNA or any increase in tCr and *m*Ins compared with healthy controls. In contrast, these MS patients showed a lower choline concentration in NAWM compared with healthy controls.

In the present work three main research questions were addressed: (**1**) Are there any metabolite concentration differences between MS patients with atypical non-lesional MRI patients and MS patients with typical white matter lesions, and are there any differences between any of these MS patients and healthy controls in white matter or in the thalamus? (**2**) Is the process of whole brain atrophy (assessed with BPF) related to metabolite concentrations in NAWM or the thalamus? (3) Are absolute metabolite concentrations in NAWM and in the thalamus of MS patients related to the severity of the disease, as assessed using the ‘Multiple Sclerosis Severity Score’ (MSSS)?

## Materials and Methods

Ethical permit was obtained from The Regional Ethics Committee in Linköping (M88-07 T93-08). Written informed consent was obtained from all subjects.

### Subjects and Data

Fifteen CDMS patients (age median, min-max 57, 32–69 years) with two or fewer T2 hyperintense white matter lesions were included in the non-lesional MS group (MRI_neg_), 20 CDMS patients (age median min/max 46, 20–66 years) fulfilling the ‘Barkhof-Tintoré criteria’ as defined in [7] were included in the MRI positive MS group (MRI_pos_), and 20 healthy control subjects (age median min/max 48, 27–72 years) were included in the control group (see Table 1).

**Table 1:** Subjects.

	Controls	MRI_neg_	MRI_pos_
Number of subjects	20	15	20
Age, [median (min-max)]	48 (27–72)	57 (32–69)	46 (20–66)
Sex [M/F]	5/15	1/14	6/14
MS type [RR/SP/PP]	–	10/3/2	12/7/1
EDSS [Median (min-max)]	–	2.50 (0.0–6.5)	3.25 (1.0–8.5)
MSSS [Median (min-max)]	–	3.65 (0.05–9.38)	3.74 (0.45–9.57)
Disease duration year [Median (min-max)]	–	16 (2–44)	13 (2–35)
Number of lesions at inclusion	–	≤2	>2
Number of MS lesions at present examination [Median (min-max)]	–	1 (0–20)	15 (4–30)
Brain Parenchymal Fraction (BPF) [Median (min-max)]	0.887 (0.787–0.938)	0.857 (0.827–0.921)	0.806 (0.719–0.869)

### MR Acquisition and Quantification of Metabolite Concentrations

**Figure 1:**
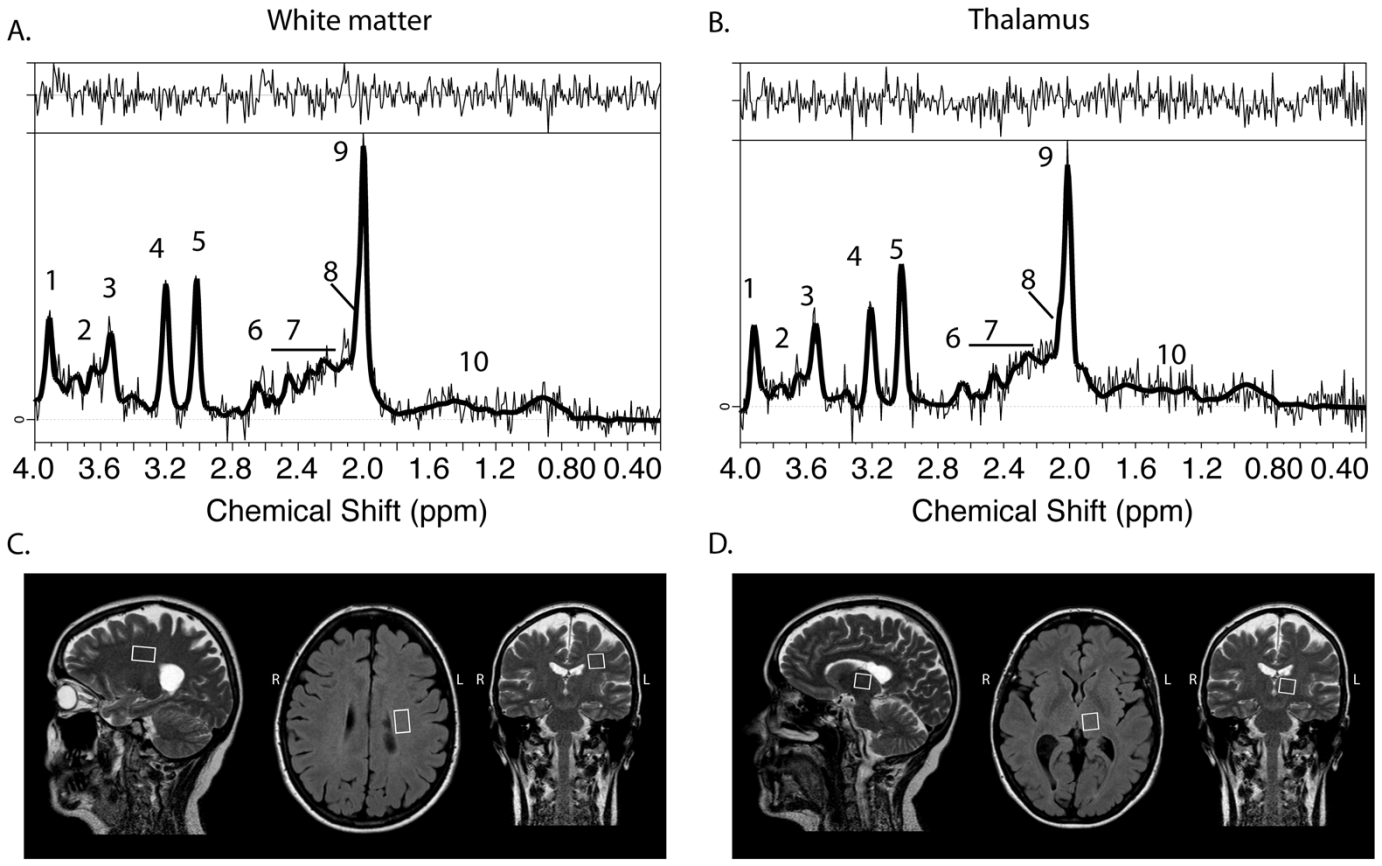
Typical spectra of white matter (A) and thalamus (B) of patient diagnosed with CDMS with no visible T2-lesions (MRIneg, female, 57 years old). Placement of MRS single volume of interest in white matter (C) and thalamus (D). Assignments of spectral resonances: 1, total Creatine (-CH2-); 2, Glutamate and Glutamine (CH-α); 3, myo-inositol; 4, total Choline ((-CH2)3); 5, total Creatine (-CH3); 6, N-acetylaspartate (CH2); 7, Glutamate and Glutamine (CH-γ/β); 8, N-acetylaspartate-glutamate (CH3); 9, N-acetylaspartate (-CH3); 10, Lactate.

All measurements were performed on a Philips Achieva 1.5 T MR scanner (Philips Healthcare, Best, the Netherlands). In summary, two MRS VOIs were placed bilaterally in the white matter and one in left the thalamus (Fig. 1). The detailed MR acquisition protocol and post-processing steps are presented elsewhere (see Cohort III in [18]).

Patient motion was monitored by acquiring a set of coronal images volumes both before and after each MRS acquisition. QMRI data was acquired using the ‘QRAPMASTER’ (also know as qMAP) sequence [7], qMRI maps of R_1_, R_2_, and C_H2O_ were calculated using SyMRI Brain Studio v. 2 software (Synthetic MR, Linköping, Sweden). SyMRI Brain Studio was also used for calculation of white matter, gray matter and CSF tissue maps [21] which were used for calculation of BPF [22]. The CSF tissue maps were also used to correct for partial volume of CSF separately in each individual MRS VOI [23]. All patients were evaluated by an experienced radiologist according to regular clinical practice.

The MRS signal was analyzed using LCModel ver 6.2-1T (S. Provencher, Canada), using an empirical basis set [24]. Both absolute concentration, which is the amount of metabolites in relation to the volume of the tissue (measured in mM), and aqueous fraction concentration, which is the amount of metabolite in relation to the amount of water in the tissue (measured in mM *aq.*) were calculated. In this report the absolute concentrations are denoted with subscript ABS and the aqueous fraction concentrations are denoted with subscript AQ. The absolute and aqueous fraction of glutamate and glutamine concentration (Glx), tNA, tCr, *m*Ins and total choline concentration (tCho) were determined. The spectral quality was assessed according to the criteria described in [25]: (1) FWHM <0.01 ppm, (2) unexplained features in the residual, (3) patient movement, (4) asymmetric line shape, and (5) outer volume ghost.

### Statistical methods

All statistical tests were performed using JMP 8.0 (SAS institute Inc., Cary NC., USA). Three different sets of mixed linear models (MLMs) were created for each metabolite, all with ‘subject’ treated as a random effect. The first set of models were used for assessing differences in metabolite concentrations between the groups and the association with age; ‘Group’, ‘Age’ and the interaction ‘Group×Age’ were all used as fixed factors. The second set of models was used to assess the association between BPF and metabolite concentrations; ‘Group’, ‘BPF’ and the interaction ‘Group×BPF’ were used as fixed factors. The third set of models was used to assess the association between MSSS and metabolite concentration in the MS patients; ‘Group’, ‘MSSS’ and the interaction ‘Group×MSSS’ were used as fixed factors. ‘Group’ was set as a nominal factor, ‘Age’, ‘BPF’ and ‘MSSS’ were set as continuous factors. If the F-test of the interaction factors resulted in a significance level of *p*<0.05, a new model was created and applied separately to each group, since significant interaction effects indicate that the independent variable affected the groups differently. The inclusion criteria for the MRI_neg_ group were based on their previous MR examinations, thus the MRI_neg_ patients could have developed lesions between their last MRI and the present MRI examination. Thus, all tests were subsequently re-evaluated, excluding patients that had developed more than two lesions.

## Results

One thalamus and one white matter MRS measurement in a control subject were excluded due to patient motion, and one thalamus MRS measurement of MRI_pos_ was excluded due to line broadening.

### Glutamate and Glutamine

The main result was that Glx_ABS_ and Glx_AQ_ in white matter were higher for both the MRI_neg_ and the MRI_pos_ group compared to the controls (Table 2). Moreover, Glx_ABS_ and Glx_AQ_ in white matter were positively correlated to MSSS (Table 3). Glx_ABS_ or Glx_AQ_ in white matter did not show any significant correlation to BPF in the MLM (with all groups, see Table 3). However, the interaction term ‘Group×BPF’ was significant and Glx_ABS_ in the white matter of the MRI_pos_ group was significantly inversely correlated to BPF (−0.11+/−0.05 mM/%, p = 0.048).

**Table 2:** Comparison of group difference and age effect on metabolite concentration in white matter and thalamus.

	Age corrected group mean concentrations						Age corrected 95% confidence intervals of mean difference									Age			
	Controls		MRI_neg_		MRI_pos_		MRI_neg_ - Controls			MRI_pos_ - Controls			MRI_neg_ - MRI_pos_			C_met_ = β*Age+Group+m_0_			
	Mean	SE	Mean	SE	Mean	SE	CI		P	CI		P	CI		P	β		SE	P
**White matter**	(N_ctrl_ = 20)		(N_neg_ = 15)		(N_pos_ = 20)		(N_neg_ = 15, N_ctrl_ = 20)			(N_pos_ = 20, N_ctrl_ = 20)			(N_neg_ = 15, N_pos_ = 20)			(N_ctrl_ = 20, N_pos_ = 20, N_neg_ = 15)			
Glx_ABS_	6.75	(0.22)	8.12	(0.27)	7.96	(0.23)	**[**0.66, 2.07**]**	***	**<.001**	**[0.57, 1.85]**	***	**<.001**	[−0.55, 0.86]		>.2	−8.00E-03		(1.20E-02)	>.2
Glx_AQ_	10.15	(0.3)	12.16	(0.36)	11.65	(0.3)	**[**1.07, 2.95**]**	***	**<.001**	**[0.65, 2.35]**	***	**<.001**	[−0.43, 1.46]		>.2	−2.40E-02		(1.60E-02)	0.16
tNA_ABS_	8.49	(0.16)	8.6	(0.2)	8	(0.17)	[−0.41, 0.63]		>.2	**[−0.96, −0.02]**	*	0.042	**[**0.08, 1.13**]**	*	0.025	−5.70E-03		(9.00E-03)	>.2
tNA_AQ_	12.75	(0.22)	12.87	(0.27)	11.78	(0.23)	[−0.58, 0.83]		>.2	**[−1.61, −0.33]**	***	0.004	**[**0.38, 1.8**]**	**	0.003	−2.00E-02		(1.20E-02)	0.12
tCr_ABS_	4.37	(0.09)	4.39	(0.11)	4.52	(0.09)	[−0.26, 0.3]		>.2	[−0.09, 0.41]		>.2	[−0.42, 0.14]		>.2	**2.00E-02**	***	**(5.00E-03)**	**<.001**
tCr_AQ_	6.55	(0.11)	6.56	(0.13)	6.64	(0.11)	[−0.34, 0.36]		>.2	[−0.22, 0.41]		>.2	[−0.43, 0.27]		>.2	**2.50E-02**	***	**(6.00E-03)**	**<.001**
mIns_ABS_	3.83	(0.12)	3.92	(0.14)	4.52	(0.12)	[−0.27, 0.47]		>.2	**[0.35, 1.02]**	***	**<.001**	**[−0.96, −0.22]**	**	0.002	**3.10E-02**	***	**(6.00E-03)**	**<.001**
mIns_AQ_	5.74	(0.18)	5.87	(0.22)	6.64	(0.18)	[−0.42, 0.7]		>.2	**[0.4, 1.42]**	***	**<.001**	**[−1.34, −0.21]**	**	0.008	**4.10E-02**	***	**(1.00E-02)**	**<.001**
tCho_ABS_	1.66	(0.05)	1.63	(0.06)	1.76	(0.05)	[−0.19, 0.13]		>.2	[−0.04, 0.25]		0.16	[−0.3, 0.02]		0.09	**6.70E-03**	*	**(2.80E-03)**	**0.019**
tCho_AQ_	2.49	(0.08)	2.44	(0.1)	2.6	(0.08)	[−0.31, 0.19]		>.2	[−0.12, 0.33]		>.2	[−0.42, 0.09]		>.2	7.90E-03		(4.40E-03)	0.08
**Thalamus**	(N_ctrl_ = 19)		(N_neg_ = 15)		(N_pos_ = 19)		(N_neg_ = 15, N_ctrl_ = 19)			(N_pos_ = 19, N_ctrl_ = 19)			(N_neg_ = 15, N_pos_ = 19)			(N_ctrl_ = 10, N_neg_ = 15, N_pos_ = 19)			
Glx_ABS_	10.62	(0.42)	10.06	(0.5)	10.91	(0.44)	[−1.88, 0.74]		>.2	[−0.94, 1.51]		>.2	[−2.18, 0.48]		>.2	2.70E-02		(2.30E-02)	>.2
Glx_AQ_	14.98	(0.57)	14.1	(0.67)	15.35	(0.59)	[−2.64, 0.88]		>.2	[−1.28, 2.01]		>.2	[−3.04, 0.54]		0.17	3.00E-02		(3.10E-02)	>.2
tNA_ABS_	8.59	(0.12)	8.91	(0.14)	8.33	(0.13)	[−0.06, 0.7]		0.09	[−0.61, 0.1]		0.15	**[0.2, 0.97]**	**	0.004	1.70E-03		(6.70E-03)	>.2
tNA_AQ_	12.09	(0.16)	12.48	(0.19)	11.73	(0.17)	[−0.11, 0.9]		0.12	[−0.83, 0.12]		0.14	**[0.23, 1.26]**	**	0.005	−6.10E-03		(8.90E-03)	>.2
tCr_ABS_	5.27	(0.12)	5.35	(0.14)	5.22	(0.12)	[−0.28, 0.44]		>.2	[−0.39, 0.29]		>.2	[−0.24, 0.5]		>.2	7.30E-03		(6.40E-03)	>.2
tCr_AQ_	7.42	(0.16)	7.49	(0.18)	7.35	(0.16)	[−0.41, 0.56]		>.2	[−0.53, 0.38]		>.2	[−0.34, 0.64]		>.2	5.50E-03		(8.50E-03)	>.2
mIns_ABS_	4.15	(0.14)	4.15	(0.16)	4.85	(0.14)	[−0.42, 0.42]		>.2	**[**0.31, 1.1**]**	***	**<.001**	**[**−1.13, −0.27**]**	**	0.002	**4.00E-02**	***	**(7.40E-03)**	**<.001**
mIns_AQ_	5.85	(0.19)	5.82	(0.22)	6.83	(0.2)	[−0.62, 0.56]		>.2	**[**0.44, 1.53**]**	***	**<.001**	**[**−1.61, −0.42**]**	**	0.001	**5.20E-02**	***	**(1.00E-02)**	**<.001**
tCho_ABS_	1.61	(0.05)	1.57	(0.06)	1.67	(0.06)	[−0.21, 0.12]		>.2	[−0.1, 0.21]		>.2	[−0.27, 0.07]		>.2	**6.90E-03**	*	**(2.90E-03)**	0.022
tCho_AQ_	2.26	(0.07)	2.19	(0.09)	2.35	(0.08)	[−0.3, 0.15]		>.2	[−0.13, 0.29]		>.2	[−0.39, 0.08]		0.18	**8.20E-03**	*	**(4.00E-03)**	0.046

Age corrected mean and standard error (SE) are presented for each group, mean absolute concentrations [mM] are presented above the aqueous fraction concentrations [mM]_AQ_. 95% confidence intervals of the mean difference between the groups are also presented. Significant effects are emphasized in **bold.** Mixed linear model of ‘Age’, and ‘Group’ are presented with estimate of linear regression coefficient β and standard error (SE).

* P<0.05,

** P<0.01,

*** P<0.001.

Results of the age effect are given in mM/year and mM_AQ_/year for the absolute concentrations and the aqueous fraction concentrations respectively. Nctrl (Number of Controls), Nneg (Number of Non-lesional MS patients) , Npos (Number of lesional MS patients).

**Table 3:** Association between metabolite concentration and brain parenchymal fraction (BPF).

	C_met_ = β* BPF+Group+m_0_			
	β	SE		P
**White matter**				
Glx_ABS_	1.26	(4.01)		>.2
Glx_AQ_	5.45	(5.54)		>.2
tNA_ABS_	−1.92	(3.05)		>.2
tNA_AQ_	0.36	(4.27)		>.2
**tCr_ABS_**	**−6.22**	**(1.63)**	***	**<.001**
**tCr_AQ_**	**−7.29**	**(2.13)**	**	**0.001**
**mIns_ABS_**	**−7.71**	**(2.43)**	**	**0.003**
**mIns_AQ_**	**−9.62**	**(3.60)**	*	**0.010**
tCho_ABS_	−0.54	(0.99)		>.2
tCho_AQ_	−0.06	(1.53)		>.2
**Thalamus**				
Glx_ABS_	−6.69	(7.80)		>.2
Glx_AQ_	−7.54	(10.47)		>.2
tNA_ABS_	0.73	(2.29)		>.2
tNA_AQ_	3.27	(3.13)		>.2
tCr_ABS_	1.45	(2.10)		>.2
tCr_AQ_	3.27	(2.79)		>.2
mIns_ABS_	−4.03	(3.33)		>.2
mIns_AQ_	−4.64	(4.60)		>.2
tCho_ABS_	−1.53	(1.02)		0.14
tCho_AQ_	−1.75	(1.39)		>.2

The mixed linear model effect of BPF on absolute concentrations [mM] are presented above the effect of BPF on aqueous fraction concentrations [mM]_AQ_. The BPF effect is presented with estimate β and standard error (SE). Significant effects are emphasized in bold.

* P<0.05,

** P<0.01,

*** P<0.001.

### N-acetylaspartate

tNA_ABS_ and tNA_AQ_ in white matter were lower for the MRI_pos_ group compared to the controls and the MRI_neg_ group (Table 2). Furthermore, tNA_ABS_ and tNA_AQ_ in the thalamus of MRI_pos_ group were significantly lower than for the MRI_neg_ group. Also, tNA_ABS_ and tNA_AQ_ in the thalamus showed a trend of being higher for MRI_neg_ compared to the controls (*p* = 0.09 for tNA_ABS_ and *p* = 0.12 for tNA_AQ_). In addition, tNA_ABS_ and tNA_AQ_ in the thalamus showed a trend of being lower in the MRI_pos_ group compared to the controls (*p* = 0.15 for tNA_ABS_, *p* = 0.14 tNA_AQ_) (Table 2).

### Creatine

tCr_ABS_ and tCr_AQ_ in white matter were positively correlated with age (Table 2). Moreover, tCr_ABS_ and tCr_AQ_ in white matter were inversely correlated to BPF (Table 3), and tCr_ABS_ in white matter was positively correlated to MSSS, while tCr_AQ_ in white matter showed a trend of positive correlation to MSSS (Table 4).

**Table 4:** Association between metabolite concentration and MS Severity Score (MSSS).

	C_met_ = β* MSSS+Group+m_0_			
	β	SE		P
**White matter**		(N = 35)		
**Glx_ABS_**	**0.16**	**(0.06)**	*	**0.019**
**Glx_AQ_**	**0.18**	**(0.08)**	*	**0.034**
tNA_ABS_	0.08	(0.06)		0.15
tNA_AQ_	0.08	(0.08)		>.2
**tCr_ABS_**	**0.06**	**(0.03)**	*	**0.041**
tCr_AQ_	0.07	(0.04)		0.09
mIns_ABS_	0.02	(0.03)		>.2
mIns_AQ_	0.01	(0.05)		>.2
tCho_ABS_	−0.03	(0.02)		0.10
tCho_AQ_	−0.05	(0.03)		0.05
**Thalamus**		(N = 34)		
Glx_ABS_	0.05	(0.13)		>.2
Glx_AQ_	0.07	(0.16)		>.2
tNA_ABS_	−0.03	(0.04)		>.2
tNA_AQ_	−0.04	(0.05)		>.2
tCr_ABS_	−0.02	(0.04)		>.2
tCr_AQ_	−0.03	(0.05)		>.2
mIns_ABS_	0.01	(0.04)		>.2
mIns_AQ_	0.02	(0.06)		>.2
tCho_ABS_	−0.03	(0.02)		0.12
tCho_AQ_	−0.04	(0.02)		0.11

The mixed linear models effect of MSSS on absolute concentrations [mM] are presented above the effect of MSSS on aqueous fraction concentrations [mM]_AQ_. The MSSS effect is presented with estimate β and standard error (SE). Significant effects are emphasized in bold.

* p<0.05,

** p<0.01,

*** p<0.001.

### Myo-Inositol

Ins_ABS_ and *m*Ins_AQ_ in white matter were significantly higher in the MRI_pos_ group compared to the controls and the MRI_neg_ group. In contrast, no concentration difference was observed between the MRI_neg_ group and the controls. Also, *m*Ins_ABS_ and *m*Ins_AQ_ in white matter were positively correlated with age. In addition, *m*Ins_ABS_ and *m*Ins_AQ_ in white matter were inversely correlated with BPF. Furthermore, *m*Ins_ABS_ and *m*Ins_AQ_ in the thalamus were significantly higher in the MRI_pos_ group compared to both controls and MRI_neg_ groups (Table 2). Also, *m*Ins_ABS_ and *m*Ins_AQ_ in the thalamus were positively correlated with age.

### Choline

No group differences in tCho in white matter were observed. tCho_ABS_ in white matter was significantly positive correlated to age, and tCho_AQ_ in white matter was showed a trend of positive correlation (as is shown in Table 3). Furthermore, tCho_ABS_ and tCho_AQ_ in the thalamus were positively correlated with age (Table 2).

Seven MRI_neg_ patients had developed more than two white matter lesions. By excluding these patients for control purposes and re-testing all data, we confirmed the significant group difference of elevated levels of Glx in MRI_neg_ patients. In contrast, significant age correlations for tCho in white matter and the thalamus were not observed. Moreover, no significant correlations of tCr_ABS_ in white matter with MSSS were detected.

## Discussion

### Glutamate and Glutamine in White Matter

The most interesting observation was that the absolute Glx_ABS_ and Glx_AQ_ concentrations were higher in NAWM of MS patients than in healthy control subjects. This was in agreement with the findings of Srinivasan *et al.*, who also found increased Glu in both acute lesions and in NAWM of MS patients [16]. Moreover, the positive correlation with MSSS indicates that Glx in NAWM may be associated with the disease progression of MS. It is also noteworthy that quantitatively determined Glx was elevated for both MRI_pos_ and MRI_neg_ patients compared to controls, indicating that elevated levels of Glx are involved in the progression of MS even in this non-lesional MS subtype. Therefore, there is a potential for Glx to be used as a marker of the severity of MS even in patients where no white matter lesions are found.

Due to the overlapping spectra of Glu and Gln (at 1.5 T), we only analyzed the sum Glx. Hence, these data do not show if it is Glu, Gln or both that were elevated in the investigated MS groups. Previous research has shown that in typical MS patients (*i.e.*, ‘MRI_pos_’) the Glu is elevated [16] or show a trend of being elevated [14], while the Gln is normal. This suggests that in our data the elevated Glx is probably due to elevated Glu. Moreover, it has been demonstrated that elevated Glu [26], [27] leads to the destruction of oligodendrocytes, which suggests a direct linkage between the elevated Glx concentrations and disease progression. Thus one might speculate that our results with elevated Glx levels in the MRI_neg_ group as well as in the MRI_pos_ group might be associated with a diffuse neurodegenerative process in MS.

### N-Acetyl Aspartate, Creatine and *myo*-Inositol in White Matter

The significantly lower absolute tNA, as well as higher *m*Ins and trend of higher tCr, in the MRI_pos_ group compared with both the MRI_neg_ group and the controls, suggest that the pathological processes underlying the common findings of lower tNA and higher *m*Ins in NAWM of MS patients are specifically linked to the presence of white matter lesions in the CNS. However, it remains to be investigated whether such lower tNA and higher *m*Ins in NAWM (1) reflect a global pathological process that will cause lesions, or (2) if they are the result of scars from old lesions, or alternatively (3) directly caused by processes associated with or emanating from active lesions through so-called ‘Wallerian’ degeneration. Wallerian degeneration has been observed in NAWM where the transected axon in a lesion is depleted within a few weeks, while in contrast the myelin in the oligodendrocytes remains intact for years [6]. Moreover, this contributing explanation agrees with the results by Vrenken *et al.* [28] who used a combination of quantitative T1-mapping and MTR-mapping and concluded that the damage found in NAWM mainly arise as a secondary result of visible lesions.

tNA is the sum of NAA and NAAG; therefore (as with Glx) the data did not reveal if it was NAA, NAAG, or both that were elevated in MRI_pos_ patients. However, tNA primarily reflects the concentration of NAA and only to a lesser extent NAAG [13]. Moreover, NAA is neuronal-specific [29] thus a reduction in neuronal density would directly lead to a reduction of tNA concentration in NAWM. It should also be noted that it has been found that reduced NAA concentration is partially reversible in acute lesions [30].

Elevated *m*Ins and tCr are often observed in NAWM [13], [14], in our data we observed elevated *m*Ins and a trend of elevated tCr concentrations. Considering that *m*Ins and tCr are a glial cell-specific marker [31] these findings suggest that the increased concentration is a result of increased glial cell density in the NAWM. Moreover, tCr_ABS_ was positively correlated with MSSS, which indicates that the process of increasing glial density is associated with MSSS.

The inverse correlation between BPF and tCr and *m*Ins indicates that the general atrophy of the brain is directly associated with locally increased tCr and *m*Ins concentrations. Moreover, the lack of correlation between BPF and tNA concentrations indicates that during the atrophy process the local tNA concentration is unchanged. Since both tCr and *m*Ins are glial cell markers [32] and tNA is a neuronal marker [29] the results indicate that during global atrophy, NAWM tissue has a constant neuronal density, while the glial density is increased. One possible model for explaining the results could be that the atrophy is caused by a reduction in both myelin amount and neuronal loss. However, the density of neurons is constant due to contraction of the tissue. Which means that the decreased BPF and constant local neuronal concentration leads to a decrease in total neuronal volume, thus BPF is a potential measure of neuronal volume. The increase in glial cell density could be caused by a proliferation of the astrocytes [6], or alternatively it could be a simple consequence of the contraction of the tissue. This model of atrophy agrees with the observation by Laule *et al.* [33], who concluded that increased R_2_ rate in MS NAWM is due to diffuse demyelination, and with Vrenken *et al.* [14] who concluded that the glial density is increased in MS NAWM.

Interestingly, the interaction term Group×BPF was not significant for tNA, *m*Ins, or tCr, indicating that the process of increased glial density with constant neuronal density in conjunction with atrophy are similar in both MS patient groups and in controls, where atrophy is normally associated with aging [34].

Moreover, the BPF were lower for the MRI_pos_ compared to both controls and MRI_neg_ (Table 1). This indicates that the elevated *m*Ins and the trend of elevated tCr in NAWM of MRI_pos_ could simply be a consequence of the contraction of the tissue. Consequently, if the tissue is contracting the lack of correlation between tNA and BPF implies neuronal loss. Furthermore, since the MRI_neg_ subjects did not exhibit a decreased BPF or reduced tNA, while the severity and disability were similar to the MRI_pos_ subjects, this indicates that the disability of MRI_neg_ patients is not directly related to the reduction of neuronal volume.

### Thalamus

No pathological metabolite concentrations in the thalamus were observed in the MRI_neg_ patient group; however, a trend of increased levels of tNA was discerned. In contrast, the MRI_pos_ group showed significantly higher *m*Ins and a trend of decreased tNA concentration in the thalamus compared with the controls. This later result agrees well with the observation by Geurts *et al.* [17], and is similar to what was found for NAWM. This suggests that it is probably the same metabolic global context that leads to the pathological concentrations of *m*Ins and tNA in NAWM and in the thalamus. In addition, whole-brain T2 lesion load has been shown to correlate well with *m*Ins concentration in the thalamus [17].

## Conclusion

Glutamate seems to be central in the pathology of MS and it is related to the progression of MS regardless of whether the patient had developed lesions at the time of MRI examination or not. In contrast, the increased glial density reflected by the increased *m*Ins concentrations and decreased neuronal density reflected by the decreased tNA concentrations in the lesion-free white matter and the thalamus were only seen in patients that had developed lesions. Moreover, the results indicated that the brain volume decreased at the same rate as the neuronal volume, while the increased glial concentration observed in MS could be due to spatial contraction of the tissue.
